# Physical activity in Sahara Moroccan hemodialysis patients

**DOI:** 10.1186/s13104-021-05460-8

**Published:** 2021-02-17

**Authors:** A. Bahadi, H. Lagtarna, S. Benbria, Y. Zajjari, D. Elkabbaj, N. Zemraoui

**Affiliations:** 1 Department of Nephrology, Dialysis and Kidney Transplantation, Military Teaching Hospital Mohammed Vth, Rabat, Morocco; 2 Department of Nephrology, Military Hospital Avicenne, Marrakech, Morocco; 3 Faculty of Medicine and Pharmacy, University Mohammed V, Rabat, Morocco; 4 Faculty of Medicine and Pharmacy, University Caddy Ayyad, Marrakech, Morocco

**Keywords:** Hemodialysis, Physical activity, Sahara, Morocco, Education

## Abstract

**Objective:**

The evaluation of physical activity for chronic hemodialysis patients is a new approach for patient global care. The objective of this work is to evaluate the physical activity in chronic hemodialysis patients and identify the risk factors associated with reduced physical activity. This is a prospective study for 6 months including 150 chronic hemodialysis patients in the Guelmim-Oued Noun Regionin Moroccan Sahara. We use Baecke's survey, translated and validated in Arabic local language. The socio-demographic, clinical, and biological data were completed during the interrogation and from the medical records of the patients.

**Results:**

The mean age of our patients was 54.6 ± 16.4 years, with male predominance (59%). Most patients have a low education level and 60% were illiterate. Hypertension was found in 54% of our patients, diabetes in 39%, and cardiovascular disease in 10% of patients. Low Physical activity was associated with gender (OR = 4.05), age (OR = 1.03) and high education level (OR = 0.2). Our work has met the various pre-established objectives, however other more specific studies must be conducted to better characterize the profile of physical activity in chronic hemodialysis patients.

## Introduction

The prevalence of end-stage renal disease (ESRD) treated with hemodialysis (HD) is increasing dramatically worldwide. In Morocco, the number of hemodialysis patients was around 7000 patients in 2008 while in December 2018 this number reached 30,000 patients according to the Moroccan society of nephrology [1, 2]. HD has completely transformed the course of chronic renal failure by improving patient survival and quality of life. However, HD is still associated with high morbidity and mortality, including the reduction of autonomy and physical activity. In this context, several studies have shown the benefit of maintaining or resuming physical activity on the health of chronic hemodialysis patients [3–6].

Physical activity is defined by the world health organization as any bodily movement produced by skeletal muscles that require energy expenditure [7]. Popular ways to be active are through walking, cycling, sports, recreation, and can be done at any level of skill and for enjoyment. This includes movements made while working, playing, doing household chores, moving around, and during leisure activities. Baecke developed in the Netherlands a questionnaire for evaluating physical activity and separating it into three representative indices: the work activity index, the sports activity index, and the index leisure activity [8]. This survey was validated as a PA assessment tool by many authors [9].

The objective of our study was to analyze the level of physical activity in chronic hemodialysis patients in Moroccan Sahara and to identify the risk factors for reduced physical activity in this area. The region Guelmim has 433,757 inhabitants, 65% of whom are urban with 9.4 inhabitants / km2 of density and it contains four provinces: Guelmim, Sidi Ifni, Tan-Tan and Assa Zag,

## Main text

### Methods

It is a prospective descriptive-analytical study spread over 6 months, from April 2019 to October 2019. This study involved a cohort of 150 hemodialysis patients in all hemodialysis centers in the Guelmim-Oued-Noune region:

- Hemodialysis Center of the Military Hospital at Guelmim (27 patients)
- Hemodialysis center of the Guelmim regional hospital (60 patients)
- Guelmim private hemodialysis center (32 patients)
- Sidi Ifni hemodialysis center (20 patients)
- Assa Hemodialysis Center (5 patients)
- Tan-Tan hemodialysis center (6 patients)

#### ***Inclusion ***criteria

we included patients on hemodialysis for more than six months, Clinically stable patients in absence of a major handicap reducing mobility (limb amputation, paraplegic, or requiring a wheelchair to move).

#### Exclusion criteria

we excluded patients with hearing impairment, Acute renal failure, and patients not consenting.

#### Data collection

The parameters likely to influence physical activity (PA) in chronic hemodialysis patients were studied. Social—demographic data were completed during the interrogation and clinical – biological data of patients were collected from the medical files: Demographic data, Socio-professional categories, clinical data, biological parameters, and dialysis data.

#### Physical activity

We assessed the physical activity of our patients using the Baecke survey consisting of 16 questions. It is self-administered, and allows in its initial form, to determine a work activity index (WAI, eight questions), a sports activity index (SAI, four questions), and a leisure activity index (LAI, four questions). An adaptation was made, which reduced the questionnaire to five questions. The answers to the first two questions determine the SAI: the combination of the intensity of the sport, its frequency of practice per week, and over the year, makes it possible to calculate the SAI. The average of the answers to the other three questions on a five-point scale allows the calculation of the leisure activity index (WAI). In our study, we studied the leisure activity of our patients (WAI). The development of the questionnaire involved several stages to adapt it to our Moroccan cultural context. The adaptation methodology we followed can be summarized as follows:

- Independent translations (from French to Moroccan Dialectal), before the summary translation which was carried out by a group of professionals representing skills in different disciplines (Nephrologists, Professors of the French language…).
- Modification of the questionnaire according to the equivalence of the Arabic Dialectal version compared to the original questionnaire on the one hand, and according to the remarks and misunderstandings of the patients who participated in the pre-test on the other hand.
- Counter-translation into French.

#### Statistical analysis

The descriptive analysis of clinical, socio-demographic, and biological data allowed the calculation of the absolute and relative frequencies for the qualitative variables, and the positioning and dispersion parameters for the quantitative variables (mean, standard deviation).

The normal distribution of variables was studied by the Kolmogorov–Smirnov test.

- In bivariate analysis, the comparison of continuous variables used the Student test and the Mann Whitney test. In the multivariate analysis, we used binary logistic regression.
- The significance threshold was retained for a p < 0.05.
- The statistical analysis was performed using IBM SPSS version 20.0 software.

The study respected patient anonymity and the confidentiality of medical information.

## Results

The average age of our 150 included patients was 54.6 ± 16.4 years, with extremes ranging from 18 to 85 years, a male predominance was noted with a sex ratio of 89 M/61F. The demographics of our study population are summarized in Table 1.

**Table 1:** Characteristics of patients at inclusion

Caracteristics	N = 150
Age (mean ± Sd) years	54.6 ± 16.4
Male n (%)	89 (59.3)
Co-morbodities	
Hypertension	82 (54.7)
Diabetes	59 (39.3)
Arteriopathy	19 (12.7)
Cardiopathy	15 (10)
Low limb fracture	11 (7.3)
Obesity	10 (6.7)
Blind	6 (4)
Depression	4 (2.7)
Neoplasm	4 (2.7)
Educational status	
Illiterate	91 (60.7)
Primary school	22 (14.7)
High school	33 (22)
University	4 (2.7)
Treatment	
Erythropoietin	74 (49.3)
Antihypertensive drug	80 (53.3)
Calcium	101 (67.3)
Dialysis data	
Months on hemodialysis (median; quartiles)	48 [24–96]
2 Sessions per week	84 (56)
KT∕V	1.33 ± 0.15
Biologic data	
Haemoglobin (mean ± ET) g∕dl	10.2 ± 2
Calcium (mean ± ET) mg∕l	87.8 ± 17.6
Phosphore (median; quartiles)	40 [17,9–51,8] [8–51]
PTHi (median; quartiles) pg∕ml	434 [240—675]
25-OH vit D2 (mean ± ET) ng∕ml	39,8 ± 14,5
C Reactive Protein (median; quartiles) mg∕l	2 [1–6,5] [1–6]

Most patients have a low level of education and 38% of patients have no job. 65% of patients live in an urban location, 30% are retired and 18% are students. The ministry of health paid hemodialysis for up to 61% of patients and others were covered by a social security system (employees, military…).

### Clinical data

12% of patients have an arteriopathy and 7% have a lower limb fracture. For comorbidities, we found hypertension in 54%, diabetes in 39%, and cardiopathy in 10%

### Physical activity

Only 19% of patients report having regular physical and/or sporting activity (walking, football). 19% of patients report having no physical activity and are mainly patients assisted in their daily activities by a third person. 63% of cases report difficulty performing significant physical efforts (running, lifting a heavy object). Regarding the means of transport used to get to hemodialysis centers, most of our patients (88%) used a vehicle (car, motorbike, etc.) while only 11% came on foot.

The calculation of physical activity indices revealed the following information: in patients who practice sports, the mean sports activity index (SAI) is 0.79 ± 0.7. Moreover, the average leisure activity index (LAI) is 2.45 ± 1.2. The average of these 2 indices is equal to 1.62 ± 0.8 which corresponds to limited physical activity, and this in the different aspects of the daily life of our HD patients.

### Low Physical activity risk factors

To search risk factors of low activity, we compare two groups: group A with low physical activity defined by a global activity index (GAI) less than 5 and group B with intermediate or high physical activity (Table 2). In the univariate analysis, we found that low activity is associated with age, female, living in a rural area, diabetes, 2 dialysis sessions by week, use of anti-hypertension drugs, and car for coming to hemodialysis center. However, after including all factors in a multivariate model only three risk factors still significant: elderly patients (OR = 1,03), female (OR = 4,05), and low education level (illiterate and primary school).

**Table 2:** Risk factors of low physical activity in univariate and multivariate analysis

Factor	Univariate analysis			Multivariate analysis		
	Low activity n = 97	Others N = 53	p	OR	IC	p
Age (mean) years	59,6	45,4	< 0.001	1.03	1–1,06	0.019
Female n(%)	46 (47.4)	15 (28.3)	0.025	4.05	1.36–12	0.012
Assurance n(%)	44 (45.4)	14 (26.4)	0.024	4.8	0.27–84	0.27
Rural area n(%)	38 (39.2)	30 (56.6)	0.017	0.32	0.07–1.38	0.12
Education ^a^ n(%)	19 (19.6)	18 (34)	0.051	0.2	0.04–0.87	0.032
Hypertension n(%)	54 (55.7)	28 (52.8)	0.8			
Diabetes n(%)	48 (49.5)	11 (20.8)	0.001	1.82	0.63–5.18	0.26
BMI < 25	82 (84.5)	15 (28.3)	0.01	5.7	0.57–57	0.137
Months on HD	69.2	58.4	0.39			
2 sessions∕ W n(%)	46 (47.4)	38 (71.7)	0.004	0.15	0.01–2.14	0.16
KT∕V	1.33	1.33	0.9			
AntiHT drugs n(%)	58 (59.8)	22 (41.5)	0.04	1.71	0.61–4.77	0.3
Depression n(%)	2 (2.1)	2 (3.8)	0.6			
Neoplasm n(%)	3 (3,1)	1 (1,9)	0.6			
Erythropoietin n(%)	48 (49.5)	26 (49.1)	0,9			
Iron drug n(%)	57 (58.8)	22 (41.5)	0.06	1.67	0.64–4.35	0.28
Calcium (mean) mg.l	89.9	83.9	0.07	1.02	0.99–1.05	0.07
Haemoglobin (mean) g.dl	10.3	9.9	0.3			
Phosphore (mean) mg.l	41,6	59	0.38			
PTHi (mean) pg.ml	527	515	0.8			
25OH vitD	39.8	39.8	0,9			
CRP (mean) mg.l	8.1	16.3	0,19			
Anemia n(%)	61 (62.9)	36 (67.9)	0.14			
Car Use ^b^ n(%)	93 (95.9)	40 (75.5)	< 0.001	2.01	0.41–9.79	0.38

^a^Education defined by college level and upper

^b^Patients using car for going to hemodialysis unit

## Discussion

It is a prospective descriptive-analytical study that required fieldwork and a trip between all the centers in the GUELMIM-OUED NOUN region. The distance traveled during the completion of this work is estimated at 648 km. The PA assessment was carried out using the Beacke survey and has been the focus of several studies which is considered to be one of the most reproducible [10, 13].

According to the literature review, the average age varies between 51.6 and 69 years. The results of our series showed an average age of 54.6 years [8, 11]. Several series in the literature report a predominance of men, notably a local Moroccan study carried out by Karimi [14] at the Al Fârâbî hospital in Oujda with a sex ratio of 45 M/38F, that of Fiaccadorie [15] carried out in Italy with a percentage of 67% male, and that of Matsuzawa [16] in Japan had a male predominance of 55%. This joins our study where the male sex is predominant with a percentage of 60%.

During our study, it turned out that the majority of our patients had a low socioeconomic level, with a thatching rate of 38%. Although the illiterate rate has decreased in Morocco thanks to programs to combat illiteracy, 61% of our population, unfortunately, was illiterate as reported by Karimi's study where the majority of patients were unemployed and illiterate [14].

Diabetes, hypertension, and cardiovascular disease are the most prevalent chronic diseases according to the literature review and were also found in our patients (diabetes 39%, hypertension 54%). In particular, diabetes, which was the leading cause of chronic kidney disease (46%) in our patients, followed by hypertension (25%).

The mean duration of hemodialysis in our study was 65.41 months. The majority of our patients are on dialysis twice a week, joining the study by Karimi [14], where most of the patients were also dialyzed twice a week, with an average duration of hemodialysis of 102.4 ± 41,9 months.

During our study, it turned out that only 19% of patients had regular physical activity. This is due to three risk factors: gender, age and low education level.

We found that females on hemodialysis in Moroccan Sahara are at high risk of low physical activity (OR 4.05). This result is supported by some studies showing that men are more active than women [17]. This can be explained by the lack of adherence to recommended physical activity which is significantly more frequent in women as found by Hornick and al [18]. The built environment also has a positive effect on physical activity in women more than men like movability index calculated based on residential density, land use mix, street connectivity, availability of public transport, and public open spaces) [19–21]. These conditions are not yet very available in such context of Sahara.

Our study shows that elderly subjects have a decreased PA compared to the youngest subjects, which joins Karimi's study. Older patients spent up to 8 h a day inactive corresponding to around 80% of their waking time [22]. This can be explained by the lack of energy and fatigue reported by several elderly respondents, in addition to the chronic pain experienced by these patients. The impact of chronic pain on the quality of life of patients is currently proven by several authors [23]. Pain is responsible for discomfort in daily activity in 67% of cases [24]. This pain can be explained by amyloidosis b2 microglobulin. The presence of amyloid deposits mainly in articular and para-articular tissues (synovial membranes, tendons, ligaments) and in bones clinically cause the appearance of joint and peri-articular pain syndromes and ductal syndromes [25].

High education level has a positive effect on PA and educated people are more physically active and pay more attention to their health [26, 27]. Varo and al (EU study) showed that people with primary level education were more sedentary than people with higher levels of education [28]. The majority of our hemodialysis patients are illiterate (61%) and the correlation of illiteracy with decreased PA can be explained in part by ignorance of the importance of regular physical activity in these patients. The study by Capitanini [29] carried out in Italy in 2014, showed that the lack of specific advice concerning physical exercise in nephrology establishments favored a sedentary lifestyle. This element should be taken into account especially when it comes to a low educated population.

## Limitations

We use a subjective means to quantify the PA of patients and The lack of financial support did not allow us to buy pedometers to better quantify the PA of our patients. We, therefore, used the Baecke survey as a tool to assess PA. We also had Incomplete biological data in some files: During operation, KT/V was mentioned in only 32 files. Only 75 files were complete. In the 75 remaining files, the phosphocalcic balance was incomplete or completely absent.

## Data Availability

Data are available any time for control and the datasets used and/or analysed during the current study available from the corresponding author on reasonable request: Bahadi Abdelaali; bahadiali@gmail.com.

## Abbreviations

ESRD: End stage renal disease
HD: Hemodialysis
PA: Physical activity
Hb: Haemoglobin
PTH: Parathormone
CRP: C reactive protein
WAI: Work activity index
SAI: Sport activity index
LAI: Leisure activity index
GAI: Global activity index
OR: Odds ratio
